# Nanofibers with diameter below one nanometer from electrospinning†

**DOI:** 10.1039/c7ra13444d

**Published:** 2018-01-26

**Authors:** Shaoju Jian, Jia Zhu, Shaohua Jiang, Shuiliang Chen, Hong Fang, Yonghai Song, Gaigai Duan, Yongfan Zhang, Haoqing Hou

**Affiliations:** Department of Chemistry and Chemical Engineering, Jiangxi Normal University Nanchang 330022 China; College of Ecology and Resources Engineering, Wuyi University Wuyishan 354300 China; College of Materials Science and Engineering, Nanjing Forestry University Nanjing 210037 China s.jiang19830913@gmail.com; College of Chemical Engineering, Nanjing Forestry University Nanjing 210037 China; Department of Chemistry, Fuzhou University Fuzhou 350108 China

## Abstract

Sub-nanometer materials have received wide attention due to their unique properties in recent years. Most studies focus on the preparation and properties investigation of the inorganic sub-nanometer materials, while there are few reports on organic especially polymeric sub-nanometer materials such as sub-nanometer fiber due to the obstacles with respect to fabricating such small nanofibers. In this work we prepare PAA nanofibers with diameters ranging from hundreds of nanometers down to sub-nanometer *via* electrospinning from a polyamic acid (PAA) with ultrahigh molecular weight. The morphologies and size of the electrospun ultrathin nanofibers are characterized using scanning electron microscopy (SEM), transmission electron microscopy (TEM), and atomic force microscopy (AFM). AFM images combined with theoretic calculations show that sub-nanometer fiber of approximate 0.17–0.63 nm only containing one molecular chain was generated *via* electrospinning from ultra-dilute PAA solutions for the first time. These quite small sub-nanometer fibers would open a new area of electrospinning and provide further explorations on the production and application of electrospun sub-nanometer fibers with single molecular chains.

## Abstract 2

Super-fine nanofibers with diameter below 1 nanometer are prepared by electrospinning from ultra-dilute solutions.

## Graphical abstract

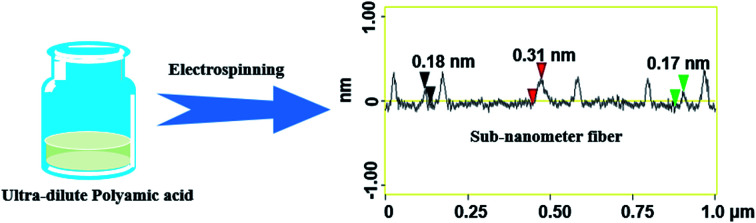

## 1. Introduction

In the past several decades, huge progress has been made in nanomaterials, and sub-nanometer materials (SNMs) is a burgeoning multi-disciplinary topic in nanoscience. The term, sub-nanometer, is used to delimit the size below one nanometer.^1^ SNMs feature molecular level size, as well as exhibit distinct properties which are different from their nano-counterparts with larger sizes such as molecule-like properties, flexibility induced behaviors and unique electronic structure. Nanocluster, graphene, and sub-nanometer metal or inorganic wires (SNWs) are representative SNMs.^2–7^ SNWs were usually synthesized by template assisted growth,^8^ oriented attached growth,^9^ ligand controlled growth,^10^ catalyst-guided growth.^11^ Most of the SNMs are made from carbon materials, metal or inorganic materials. However, there are few reports regarding the one dimensional sub-nanometer polymeric fibers. Generally, sub-nanometer polymeric fibers refer to the one dimensional structure with ultrahigh aspect ratio with the diameter ranging from several angstroms to dozens of angstroms. From the theoretic point of view, such sub-nanometer polymeric fibers can only contain several polymer chains or even single macromolecular chain. These sub-nanometer fibers consisting of only one polymer chain have potential applications for size standards or molecular mass. The behavior of sub-nanometer fibers is expected to be different from bulk polymer materials or even conventional nanomaterials. Direct behavioral information of sub-nanometer fibers will inspire the appearance of new theories in polymer physics and also be very useful for the development of molecular devices based on high performance polymer materials. However, it was difficult to obtain its single molecular level information, *e.g.* mechanical, electrical and magnetic properties due to the obstacle toward fabricating such small nanofiber. Ultrathin nanofibers with the diameter of less than 100 nm can be produced by phase separation,^12^ self-assembling,^13^ sea-island method,^14^ template synthesis,^15^ electrospinning,^16^ and bubble-electrospinning.^17^ For example, crystalline nanofibers of linear polyethylene with the diameter of ranging from 30 to 50 nm was obtain by freeze-drying the polymeric mass with ultra-high molecular weight polyethylene.^18^ Fu Renchun *et al.* fabricated pure PANI nanofibers with high electrical conductivity and a diameter of 40–50 nm using ethyl cellulose as the template.^19^ Kazuhiro Nakata *et al.* prepared continuous PET nanofibers with the diameter of 39 nm by sea-island-type conjugated meltspinning and laser-heated flow drawing method.^20^ Yang *et al.* successfully produced ultrafine PVA nanofibers with diameter of 20 nm or less through bubble-electrospinning method.^21^ In our previous work, continuous nylon-4,6 nanofiber with diameter of 1.6 nm had been successfully yielded by electrospinning, which only contains tens of polymer chains in theory.^22^ To the best of our knowledge, it is the smallest nanofiber which has been reported until now. Electrospinning is a straightforward and simple method to produce polymer nanofibers with diameters ranging from several micrometers to several nanometers, by forcing polymer liquids (solution or melt) through a spinneret with an electric field.^23–25^ Electrospinning occurs when the electrical forces form the electrostatic repulsion at the surface of a polymer solution or melt overcome the surface tension and creates an electrically charged jet out of the pipette.^26^ In traditional electrospinning, researchers are always restricted by the mechanism of electrospun fiber formation requiring highly entanglements of macromolecules in a certain viscous polymer solution.^27–29^ Although electrospinning is a facile technology to produce ultrathin nanofibers, till now, to the best of our knowledge, it is still a great challenge to prepare sub-nanometer fibers by electrospinning.

In this work, we break through the constraint of the formation mechanism of traditional electrospun fibers and proposed the concept to produce sub-nanometer fibers by electrospinning ultra-dilute polymer solution with weak macromolecular entanglement. *Via* electrospinning an ultra-dilute solution (0.03–0.01 wt%) from a polyamic acid (PAA) with ultra-high molecular weight (*M*_n_ > 10^6^ g mol^−1^), the sub-nanometer fibers with diameter approximate 0.17–0.63 nm were achieved. Further molecular simulation suggests that such small nanofiber contains even only one molecular chain. This is the first time to produce single molecular chain-based sub-nanometer fibers from electrospinning, and this discovery is expected to open a new insight to the preparation of single molecular chain-based sub-nanometer fibers and future investigations on their properties and applications.

## 2. Experimental

### 2.1 Preparation of polymer solution for electrospinning

The PAA solution (8.74 wt%) for electrospinning, was synthesized from equimolar ratio of dianhydride BPDA and BPA by a polycondensation in DMAc at −5 °C with intense mechanical stirring for 24 h.^30,31^ The macromolecular structure of PAA was shown in Fig. S1.† The intrinsic viscosity ([*η*]) and the average molecular weight (*M*_w_) were 5.4 dL g^−1^ and 1.09 × 10^6^ g mol^−1^, respectively.

### 2.2 Electrospinning

The solutions for electrospinning were prepared by diluting the as-prepared PAA solution (8.74 wt%) with different concentrations from 6 wt% to 0.01 wt%. 0.1 wt% of DTAC with respect to DMAc was added to increase the electrical conductivity of the solutions when the PAA solution concentration was below 2 wt%. Polymer solution in DMAc was held in a syringe with an internal diameter of about 12.5 mm with a needle (internal diameter of 0.45 mm). The electric fields were 200 kV m^−1^, by applying a positive 30 kV and a negative 10 kV electrical potential between a spinneret and a collector. The electrospun nanofibers were collected with 20 cm collecting distance by copper grids with holy carbon films for SEM and high-resolution TEM measurement and freshly cleaved mica for AFM characterization respectively. All the samples were dried at 80 °C for 8 h in vacuum to remove the residual solvent.

### 2.3 Measurements and characterization

The intrinsic viscosity measurements were measured at 25 °C using an ubbelohde capillary viscometer. The morphologies of polymer nanofibers were examined by scanning electron microscopy (SEM), transmission electron microscopy (TEM), and atomic force microscopy (AFM). SEM measurements were performed with a JSM-6701F microscope (JEOL Ltd, Japan) at an acceleration voltage of 5 kV. TEM images were obtained using a JEM 2100 microscope (JEOL Ltd, Japan) and a Technai-12 microscope (FEI, USA) with an operation voltage of 200 kV and 120 kV, respectively. AFM measurement was carried out with a Multimode V8 instrument equipped with a NanoScope V controller (Bruker Corporation, Germany) and a standard silicon cantilever (OTESPA model) with a length ranging from 140 to 180 μm and typically resonance frequency of about 300 kHz. The tip radius is about 7 nm. All AFM images (512 × 512 pixels) were obtained with a scan rate of 1.0 Hz and scan angle of 0° in tapping mode at room temperature under ambient conditions. Offline images were only simply flattened by Nanoscope III 5.12r2 software without any further process.

## 3. Results and discussion

### 3.1 Effect of concentration and conductivity on the fiber morphology

Diameters of electrospun fibers can be tuned by changing the concentrations of polymer solutions.^32–34^ The formation of macromolecular chain entanglements has been acknowledged as the primary effect for the fiber formation. According to the concentration dependence of viscosity of linear polymers in good solvents, Colby *et al.* identified four different concentration regimes including dilute, semidilute unentangled, semidilute entangled, and concentrated regimes.^35^ The critical chain overlap concentration (c*), is the boundary between the dilute and the semidilute concentration regimes. The entanglement concentration (*c*_e_) is the crossover concentration between the semidilute unentangled and semidilute entangled regimes. Physically, *c*_e_ is defined as the point at which a significant overlap of polymer chains topologically constrains chain motion, causing entanglement couplings. Guopta *et al.* defined the value of *c*/c* for the different solution regimes, dilute regime (*c*/c* < 1), semidilute unentangled regime (1 < *c*/c* < 3), semidilute entangled solutions (3 < *c*/c* < 6), relatively concentrated solutions (*c*/c* > 6), and the entanglement concentration (*c*_e_) (*c*/c* ∼ 3).^36^

**Fig. 1:**
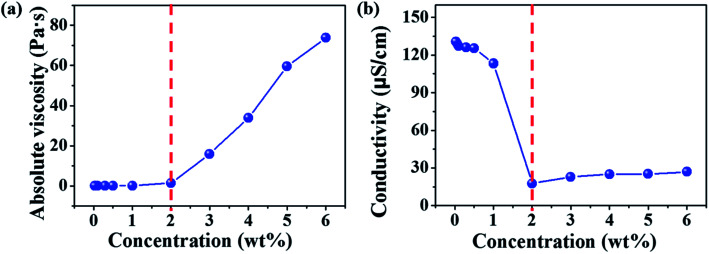
Absolute viscosity (a) and electrical conductivity (b) of polymer solutions with different concentrations (0.1 wt% of DTAC with respect to the DMAc solvent was added in to the solutions when the concentrations were <2 wt%).

Electrospinning of dilute, semidilute unentangled, semidilute entangled and concentrated polymer solutions resulted in the formation of only polymer droplets or beads, a mixture of polymer droplets and beaded fibers, beaded fibers, uniform and beaded free fibers, respectively. In general, the critical chain overlap concentration (c*) is frequently defined by the reciprocal of the intrinsic viscosity,^36,37^ 1/[*η*]. Hence, according to Guopta's study, c* of the PAA solution is about 0.19 wt%, and the concentrations used to electrospinning in this study can be divided into four categories: dilute regime (0.01–0.1 wt%), semidilute unentangled regime (0.3 wt% and 0.5 wt%), semidilute entangled (1 wt%), and concentrated regime (2–6 wt%). The absolute viscosity and electrical conductivity of the above polymer solutions with different concentrations were summarized in Table S1† and plotted in Fig. 1. It can be seen that the absolute viscosity of polymer solutions increases with the concentration increased. In concentrated regime (2–6 wt%), polymer chains entangled with each other, therefore the interactions between polymer chains increase, resulting in significant increasing in viscosity as the solution concentrations increase. While polymer chains are isolated by solvent and act as separate coil in other three regimes (<2 wt%), so increasing solution concentrations do not affect the viscosity obviously. The electrical conductivity of polymer solution is dependent on both the concentration and the additional organic salt in the solution because the charge density of the polymer solution increases from the ions ionized mainly from DTAC. The electrical conductivity of polymer solution decreases with the increasing of concentration when the solution concentrations below 2 wt% because the amounts of DTAC (0.1 wt% of the solvent) increase with the decreased solution concentrations.

**Fig. 2:**
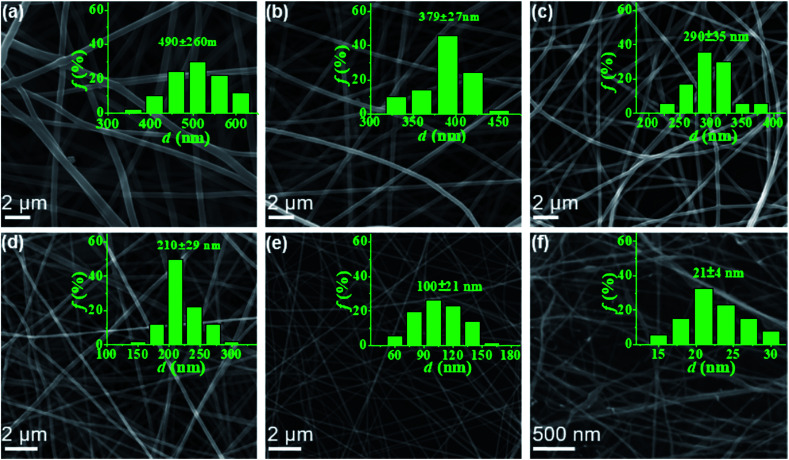
SEM micrographs of PAA nanofibers obtained from PAA solutions of 6 wt% (a), 5 wt% (b), 4 wt% (c), 3 wt% (d), 2 wt% (e), and 1 wt% (f), respectively. The insets showed the diameter distribution of the corresponding fibers.

In this work, electrospun nanofibers with different diameters can be obtained by adjusting the concentrations of PAA solution. Fig. 2 shows SEM images of nanofibers with corresponding diameter distributions from PAA solutions with various concentrations. For the 6–2 wt% solutions in concentrated regime, the polymer chains in solutions entangle with each other, leading to the smooth and uniform electrospun PAA nanofibers without any beads on the fibers (Fig. 2a–e). As expected, the average diameters of nanofibers decreased dramatically from 490 ± 60 to 100 ± 21 nm when the concentrations decreased from 6 wt% to 2 wt%. When the concentration decreased to semidilute entangled regime (1.0 wt%), some beaded fibers but with very thinner diameters of 21 ± 4 nm were generated (Fig. 2f). These beaded fibers could be because of the insufficient intermolecular entanglements between polymer chains to overcome the capillary instability. Further investigation by TEM images (see Fig. S2†) on the fiber morphology and fiber diameter was well agreement with the results obtained by SEM analysis (Fig. 2).

**Fig. 3:**
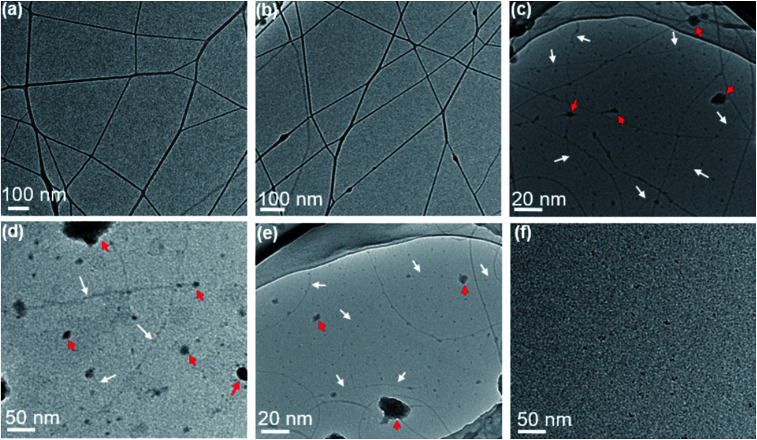
TEM images of PAA nanofibers obtained from PAA solutions of 0.5 wt% (a), 0.3 wt% (b), 0.1 wt% (c), 0.06 wt% (d), 0.03 wt% (e) and 0.02 wt% (f). The insets of (a) and (b) presented the diameter distribution of the corresponding fibers. White arrows indicate the sub-nanometer fibers and red arrows indicate the polymer droplets.

Further decreasing the concentration of PAA solution in semidilute unentangled regime led to a mixture of ultra-thin nanofibers, beaded fibers and few polymer droplets (Fig. 3a and b), and the average fiber diameter further decreased. The sub-nanometer fibers with diameter less than 1 nm (indicated by white arrows, Fig. 3c and e) could be achieved when the concentration of PAA solutions was in the dilute regime of 0.1, 0.06 and 0.03 wt%. The smallest measured diameter of the sub-nanometer fiber by TEM was 0.98 nm (Fig. 3d). In this regime, a few large polymer droplets simultaneously (indicated by red arrows, Fig. 3c–e) were observed due to the insufficient chain overlap between the chains. Unfortunately, the morphology and size of the sub-nanometer fiber with thinner diameter could not be precisely measured even by electron microscopy because the spatial resolution of SEM and TEM can hardly reach that regime (several faint fiber images indicated by white arrows in Fig. 3c–e). In addition, when the concentration further decreased to 0.02 wt%, it is difficult to observe sub-nanometer fibers, which could be because of the resolution limitations of TEM caused by holy carbon films covered on the copper grid (Fig. 3f). Therefore, it is highly required that a technique can be used for precise determination on the morphology and fiber diameters for the samples, which were prepared from ultra-dilute PAA solutions by electrospinning.

### 3.2 Sub-nanometer PAA fibers by AFM

**Fig. 4:**
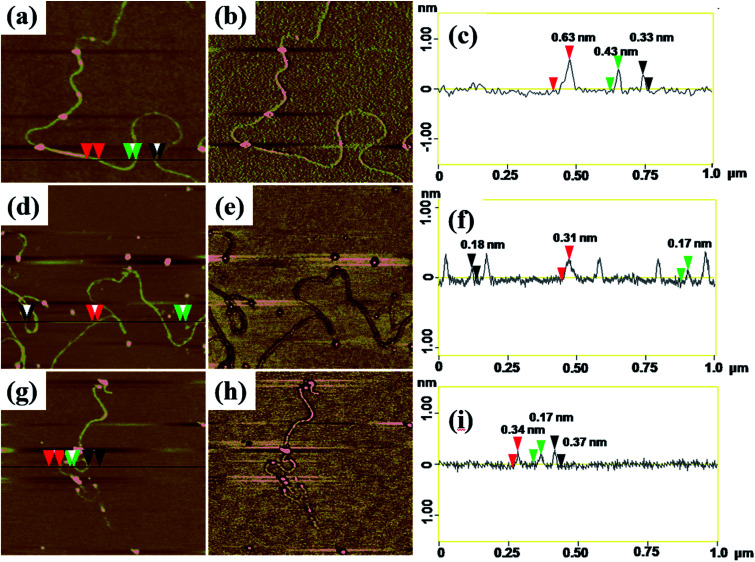
Height images and corresponding phase images and section analysis of (a)–(c) nanofibers obtained from 0.03 wt% PAA solution; (d)–(f) nanofibers obtained from 0.02 wt%; (g)–(i) nanofibers obtained from 0.01 wt% PAA solution, scale sizes: 1 × 1 μm, *z*-scales: (b) 3°, (e) 2°, (h) 2°.

AFM with tapping mode can greatly reduce the irreversible destruction of sample surfaces, and has been widely utilized to study the structure and morphology of samples at molecular or even atomic resolutions in three dimensions.^38,39^ For AFM characterization, electrospun fibers obtained from PAA solutions with various concentrations were collected by using newly exfoliated mica. The morphologies of the fibers were shown in Fig. S3† and 4. The corresponding fiber diameters measured by AFM height images were shown in Table S1.† The fiber diameter was well consistent with the results obtained by TEM and SEM analysis when the PAA concentration in the range of 0.3–5 wt%. When the PAA concentration came to 0.1–0.01 wt%, continuous sub-nanometer fibers with diameter of less than 1 nm could be achieved by electrospinning. It is well known that phase images in AFM were generated as a consequence of variations in material properties, such as viscoelasticity, friction, and adhesion.^40,41^ In this experiment, phase images together with height images were used to discriminate the topography of the single-molecule chains on the surface of sub-nanometer fibers. It is evident that phase images can produce very high material contrast of fine structures unveiling more details of the morphology that barely can be seen in height images as shown in Fig. 4. From the cross-section analysis (Fig. 4c, f and i), it can be seen that the diameter of nanofibers electrospun from 0.03 wt% PAA solution can be small to 0.3–0.7 nm; the diameters of nanofibers electrospun from 0.02 wt% and 0.01 wt% solutions can be thin to 0.17–0.4 nm. The different diameters at different position of the same sub-nanometer fiber could be attributed to the different macromolecular conformation along the fibers. The width of sub-nanometer fiber is approximate 50 nm due to the convolution effect.^42,43^

### 3.3 Effect of *c*/c* on fiber diameter

**Fig. 5:**
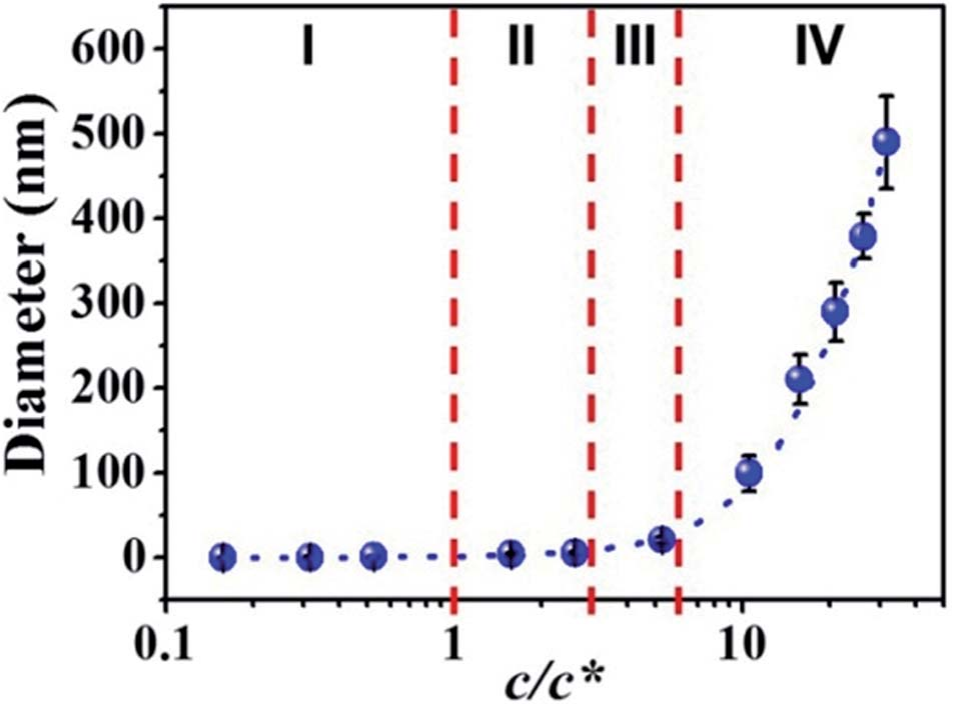
Diameters of electrospun PAA fibers from different concentration regimes. I: dilute regime (0.01–0.1 wt%); II: semidilute unentangled regime (0.3 wt% and 0.5 wt%), III: semidilute entangled regime (1 wt%), and IV: concentrated regime (2–6 wt%). Dilute regime (*c*/c* < 1), semidilute unentangled regime (1 < *c*/c* < 3), semidilute entangled solutions (3 < *c*/c* < 6), relatively concentrated solutions (*c*/c* > 6).

By correlation the fiber diameters to the solution concentrations, the *c*/c* dependent fiber diameters was plotted in Fig. 5. The relationship between fiber diameter (*Y*) and *c*/c* (*X*) in this study can be fitted by cubic model (dashed blue line in Fig. 5) with the eqn (1):

1 *Y* = *a* + *bX* + *cX*^2^ + *dX*^3^

where *a*, *b*, *c*, and *d* have values of 0.61948, 0.08154, 0.92149 and −0.01383 (Table S2†), respectively. When the *c*/c* > 6, the solution concentration was in the concentrated regime (IV) and the fiber diameter *Y* increased dramatically. In the semidilute entangled regime (III) and semidilute unentangled regime (II), the effect of solution concentration on the fiber diameter became smaller and smaller. When the solution concentration shifted into dilute regime (I), it had a tiny effect on the fiber diameters. In this regime (I), the fiber diameter decreased in the range of sub-nanometer, where fibers composed of single molecular chain or several molecular chains could be obtained.

### 3.4 Model of repeat unit of PAA molecules

To investigate the molecular conformation in such sub-nanometer fibers, theoretical calculations were performed. The structural optimizations for macromolecules of PAA were performed using the projector-augmented wave (PAW) formalism of density functional theory (DFT), as implemented in the Vienna *ab initio* simulation package (VASP),^44–46^ and the Perdew–Burke–Ernzerhof (PBE) type exchange-correlation.^47^ The kinetic energy cutoff for the plane-wave expansion was set to 500 eV, and the effects of spin polarization were considered. The repeat unit of single PAA chain contains 28 C atoms, 2 N atoms, 6 O atoms and 18 H atoms in the systems with the dimension of 30 × 30 × 21.86 Å. The minimum distance between the sidewall of the individual macromolecules of PAA and its periodic images is greater than 24.0 Å, which is large enough to avoid the interaction between the neighboring single PAA chains. And a (1 × 1 × 3) Monkhorst–Pack was used for the *k*-point sampling. The role of the van der Waals (vdW) interaction was investigated using DFT-D2 method including the pairwise force field implemented by Grimme.^48^

### 3.5 Simulation of PAA molecules in sub-nanometer fibers

**Fig. 6:**
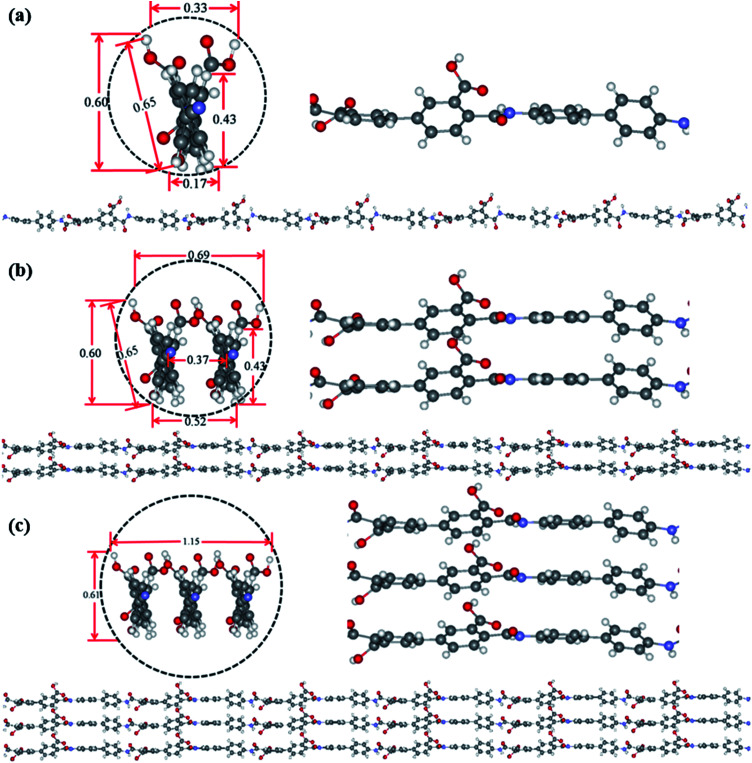
Optimized ground state structures for individual (a), two (b) and three (c) molecules of PAA molecular chain (cross-section, side view, and molecular chain). The labelled diameter are given in nm.

Theoretical simulation was performed to investigate the stack of PAA macromolecules in the sub-nanometer fibers. Fig. 6 and S4† present the molecular structure models for individual, two and three molecules of PAA molecular chain (cross-section, side view, and molecular chain). The smallest sub-nanometer fibers composed of only one PAA molecular chain. According to the simulation, the single PAA molecule should possess a diameter ranging from 0.17 to 0.65 nm. As shown in Fig. 6a, when the individual PAA molecule is parallelly anchored onto the substrate, the smallest diameters of around 0.17–0.33 nm could be obtained. If the individual PAA molecule is vertically anchored onto the substrate, the calculated diameters are ranging from 0.43 nm to 0.65 nm. This simulation on the diameter is well agreed with the measurement by AFM in Fig. 4d–i (0.17–0.37 nm). Therefore, we can speculate that the PAA nanofiber observed at AFM images as shown in Fig. 4d–i should contain only one individual PAA molecular chain. The PAA fibers with two molecular chains were also studied by the simulation. The model in Fig. 6b showed the best thermodynamical stability of the two PAA molecules while Fig. S4a and b† presented the models with energy 0.37 eV and 0.74 eV higher than the most stable model in Fig. 6b, respectively. For the stable structures in Fig. 6b and S4a,† the diameter of the two stacked PAA molecules was in the range of 0.5–0.7 nm, either for parallel or vertical model. This size is consistent with the diameters results of sub-nanometer by AFM measurement in Fig. 4a–c (0.33–0.63 nm). The simulated diameter around 0.7 nm was also detected when the individual PAA molecule is vertically anchored onto the substrate (Fig. 6a), but this vertical mode should be not stable. Thus, the simulation and analysis on two PAA molecules proved that the sub-nanometer fibers with diameter in the range of 0.5–0.7 nm by AFM should contain only two PAA molecules. Further simulation of stack of three PAA molecules was presented in Fig. 6c and S4c,† which shows the most stable configuration and the model with energy 0.79 eV higher, respectively. In these two cases, the observed sizes of the three PAA chains were in the range of 0.61–1.51 nm, which agreed with the diameter results from electrospinning of 0.1 wt% and 0.06 wt% PAA solutions (Table S1,† 0.93 ± 0.20 nm, and 0.88 ± 0.20 nm). The simulated diameter more than 1 nm was also observed for the two stacked PAA molecules chains in Fig. S4b† with vertical mode, but this vertical mode was usually not stable and could not obtained in the fibers. Therefore, these fibers from 0.1 wt% and 0.06 wt% PAA solutions with diameter around 1 nm should compose of three PAA molecules.

### 3.6 Formation of sub-nanometer PAA fibers

It is well known that nanofiber properties can be effect by numerous parameters such as applied voltage, needle to collector distance, initial jet radius, relaxation time, and solution properties like viscosity, polymer concentration, conductivity and solvent.^26,33,49,50^ Polymer chain entanglements are one of many parameters that can significantly affect nanofiber formation during the electrospinning process. At high concentration, chain entanglement behaves in a similar manner as chemical cross links, significant overlap of neighboring chains occurs, so uniform nanofibers can be produced *via* electrospinning. In ultra-dilute polymer solutions, the randomly distributed chains are separated and there is no topological constraint or polymer chains entanglement in initial electrospun solution. From the perspective of the fiber formation by traditional electrospinning, in dilute regime, application of voltage results in electrospraying or bead formation primarily due to Rayleigh instability.^28^ However, in this paper, we successfully fabricated sub-nanometer PAA fibers *via* electrospinning of ultra-dilute PAA solutions (concentrations below 0.1 wt%). The formation mechanism of traditional electrospinning had been investigated in detail by Reneker group^51,52^ and other researchers.^53,54^ Reneker *et al.* found that the detail motion path of the continuous jet is complex and the draw ratio of the jet under the applied electrical forces can reach up to 10^5^ accounting for evaporation of the solvent.^51^ When longitudinal strain rate multiplied by the conformational relaxation time of the molecule is greater than 0.5, the random coil begins to transform to a stretched macromolecule and the macromolecular coils are likely to be stretched along the axis of the jet, which led to the decrease of cross-sectional area of the jet as much as 10^5^. Presumably, in the process of electrospinning of ultra-dilute solution, when the ejected jet flows far away from the tip, the solvent evaporates rapidly and the concentration of jet increases considerable. Subsequently, weak topological constraint between polymer chains and gradient viscosity occurs. It gives the macromolecular chains a chance to entangle with each other. When the chain entanglements can sufficiently stabilize the electrospinning jet, sub-nanometer fibers can be yielded, otherwise the jet break up into little droplets.

## 4. Conclusions

In this work, sub-nanometer fibers containing only one or two polymer molecular chains were successfully prepared for the first time by electrospinning from ultra-dilute PAA solution. The fiber diameters are greatly influenced by the solution concentration and conductivity. The PAA solutions in dilute regime (0.01–0.1 wt%) with electrical conductivity ∼120 μS cm^−1^ can lead to the electrospun fibers in sub-nanometer. SEM and TEM are difficult to observe such small fibers due to the sensitivity of polymer molecules to electron beam and the spatial resolution of electron microscopy. AFM with tapping mode are useful to observe the morphology and measure the fiber diameter of such sub-nanometer PAA fibers. From AFM measurements, the smallest sub-nanometer PAA fibers possessed diameters in the range of 0.17–0.63 nm. Further theoretic simulations suggest that such sub-nanometer fibers contain only one or two PAA molecular chains. Future developments on such electrospun sub-nanometer fibers could be their physical properties and applications.

## Conflicts of interest

There are no conflicts to declare.

## Supplementary Material

RA-008-C7RA13444D-s001
