# Supplementary material for: Nanofibers with diameter below one nanometer from electrospinning
Source: RSC Adv. 2018 Jan 26;8(9):4794–802. doi: 10.1039/c7ra13444d (PMC9077775; doi:10.1039/c7ra13444d)
Supplement: RA-008-C7RA13444D-s001 [file RA-008-C7RA13444D-s001.pdf]

Electronic Supplementary Material (ESI) for RSC Advances.
This journal is © The Royal Society of Chemistry 2018

Supplementary Information

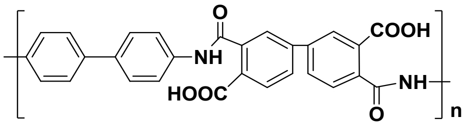

Fig. S1 Macromolecular structure of PAA.

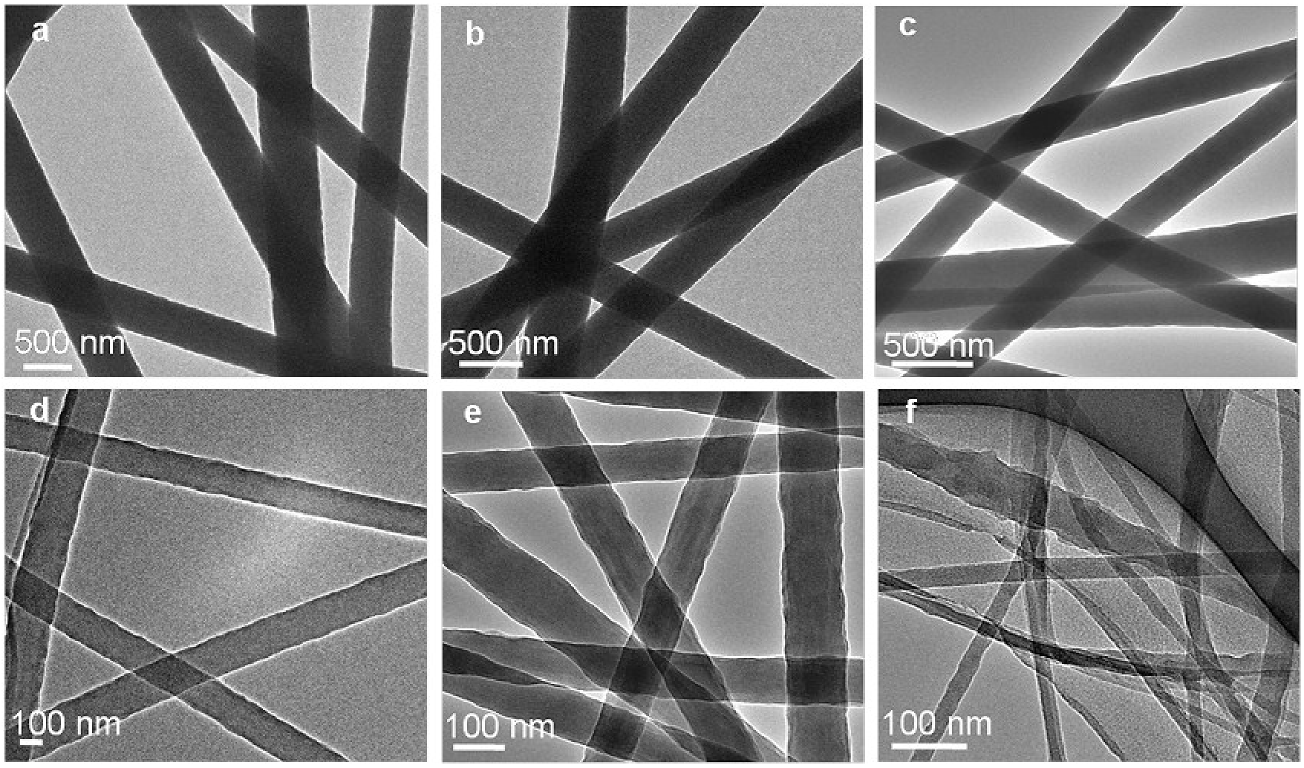

Fig. S2 TEM images of PAA nanofibers obtained from PAA solutions of 6 wt% (a), 5 wt% (b), 4 wt% (c), 3 wt% (d), 2 wt% (e), and 1 wt% (f), respectively.

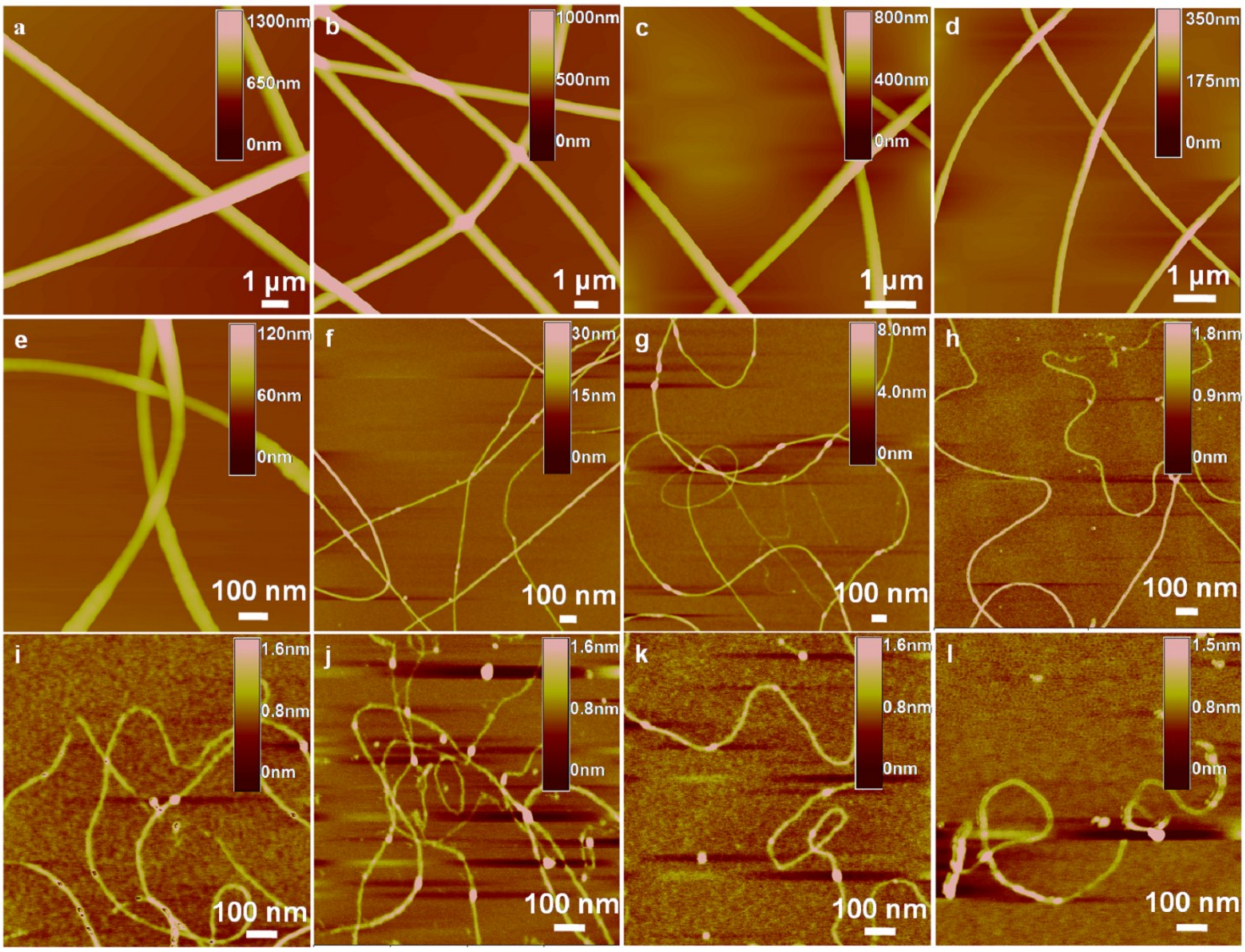

Fig. S3 AFM height images of PAA nanofibers obtained form 5 wt% (a), 4 wt% (b), 3 wt% (c), 2 wt% (d), 1 wt% (e), 0.5 wt% (f), 0.3 wt% (g), 0.1 wt% (h), 0.06 wt% (i), 0.03 wt% (j), 0.02 wt% (k) and 0.01 wt% (l) by weight concentration from PAA solution.

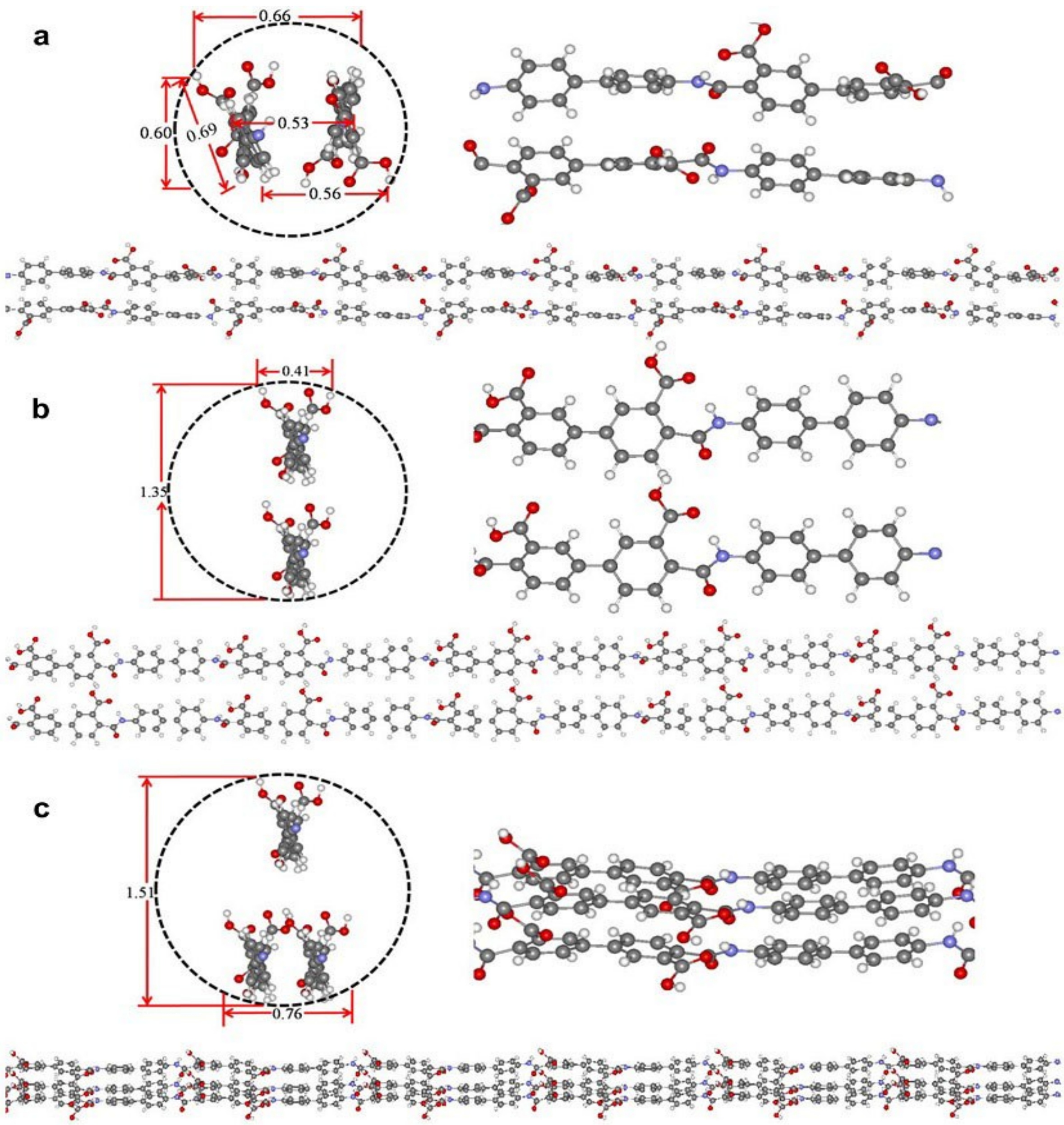

Fig. S4 Molecularmodelsfor two (a, b) and three (c) molecules of PAA molecular chain (cross-section, side view, and molecular chain), the labelled diameters are given in nm.

Table S1. Average diameters of PAA nanofibers electrospun at different concertrations and physical properties of polymer solutions.

PAA	c/c*	Diameter (nm)	Absolute	Conductivity

concentration (wt%)			viscosity(pa.s)	(μs/cm)
6	31.58	490±60	73.70	27.0
5	26.32	379±27	59.50	25.2
4	21.05	290±35	33.90	25.1
3	15.79	210±29	15.96	22.8
2	10.53	100±21	1.46	17.7
1	5.26	20±4	0.19	113.2[1]
0.5	2.63	5.83±4.54	0.17	138.3[1]
0.3	1.59	3.84±1.15	0.16	109.6[1]
0.1	0.53	0.93±0.20	0.16	127.1[1]
0.06	0.32	0.88±0.20	0.10	129.5[1]
0.03	0.16	0.49±0.20	0.05	130.5[1]
0.02	—	0.18; 0.31; 0.17	—	—
0.01	—	0.34; 0.17; 0.37	—	—

[1] 0.1wt% DTAC with respective to solvent was added into the polymer solution.

Table S2. Parameters for the fitting of Figure 5.

**Model**<br>**Equation**	**Cubic**<br>**$Y=a + bX + cX^2 + dX^3$**<br>**Value**	**Standard Error**
a	0.61948	0.19128
b	0.08154	0.51573
c	0.92149	0.1407
d	-0.01383	0.00478
